# Identification of novel functional brain proteins for treatment-resistant schizophrenia: Based on a proteome-wide association study

**DOI:** 10.1192/j.eurpsy.2023.20

**Published:** 2023-04-14

**Authors:** Wenming Wei, Huijie Zhang, Bolun Cheng, Xiaoyue Qin, Dan He, Na Zhang, Yijing Zhao, Qingqing Cai, Sirong Shi, Xiaoge Chu, Yan Wen, Huan Liu, Yumeng Jia, Feng Zhang

**Affiliations:** ^1^Key Laboratory of Trace Elements and Endemic Diseases of National Health and Family Planning Commission, Key Laboratory of Environment and Genes Related to Diseases of Ministry of Education of China, School of Public Health, Health Science Center, Xi’an Jiaotong University, Xi’an, Shaanxi, China; ^2^Department of Psychiatry, The First Affiliated Hospital of Xi’an Jiaotong University, Xi’an, Shaanxi, China

**Keywords:** Human brain proteins, inflammation, lipid oxidation, mitochondria, proteome-wide association study, treatment-resistant schizophrenia

## Abstract

**Objective:**

Genetic approaches are increasingly advantageous in characterizing treatment-resistant schizophrenia (TRS). We aimed to identify TRS-associated functional brain proteins, providing a potential pathway for improving psychiatric classification and developing better-tailored therapeutic targets.

**Methods:**

TRS-related proteome-wide association studies (PWAS) were conducted on genome-wide association studies (GWAS) from CLOZUK and the Psychiatric Genomics Consortium (PGC), which provided TRS individuals (*n* = 10,501) and non-TRS individuals (*n* = 20,325), respectively. The reference datasets for the human brain proteome were obtained from ROS/MAP and Banner, with 8,356 and 11,518 proteins collected, respectively. We then performed colocalization analysis and functional enrichment analysis to further explore the biological functions of the proteins identified by PWAS.

**Results:**

In PWAS, two statistically significant proteins were identified using the ROS/MAP and then replicated using the Banner reference dataset, including CPT2 (*P*_PWAS-ROS/MAP_ = 4.15 × 10^−2^ and *P*_PWAS-Banner_ = 3.38 × 10^−3^) and APOL2 (*P*_PWAS-ROS/MAP_ = 4.49 × 10^−3^ and *P*_PWAS-Banner_ = 8.26 × 10^−3^). Colocalization analysis identified three variants that were causally related to protein expression in the human brain, including *CCDC91* (PP4 = 0.981), *PRDX1* (PP4 = 0.894), and *WARS2* (PP4 = 0.757). We extended PWAS results from gene-based analysis to pathway-based analysis, identifying 14 gene ontology (GO) terms and the only candidate pathway for TRS, metabolic pathways (*all P <* 0.05).

**Conclusions:**

Our results identified two protein biomarkers, and cautiously support that the pathological mechanism of TRS is linked to lipid oxidation and inflammation, where mitochondria-related functions may play a role.

## Introduction

Treatment-resistant schizophrenia (TRS) refers to approximately one-third of schizophrenia patients who do not adequately alleviate their psychotic symptoms despite standard antipsychotic treatment [1]. Patients with TRS have higher rates of unemployment, poorer quality of life, and poorer social and occupational functioning than individuals who respond to treatment [2]. Clozapine is the only antipsychotic recommended for TRS, which is effective in about 60% of cases [3] and improves indicators of morbidity and mortality [1]. Nevertheless, studies have shown that identification difficulties with TRS led to delays in clozapine prescription, which in turn was associated with reduced responsiveness of patients to clozapine [4]. This makes early identification of TRS critical and the ascertainment of biomarkers of TRS a priority for the field of schizophrenia research.

Evidence suggests that earlier age of schizophrenia onset is a robust predictor of TRS, and that male gender, autumn/winter birth, poor premorbid functioning, and rural upbringing may also contribute [5]. However, a gap in the literature exists in the genetics of TRS, particularly biomarkers. To date, there is considerable heterogeneity in the genetic findings associated with TRS. A family history of schizophrenia is likely linked to developing TRS [6]. Candidate gene research investigating the involvement of specific targets in TRS mostly clustered around the serotoninergic and dopaminergic systems, as well as on systems involved in oxidative stress and inflammation [7]. Conversely, a small sample study of TRS, defined by American Psychiatric Association criteria, did not find any significant association among the 384 candidate loci [8]. The collective interpretation of these results is made difficult by the slightly different recruitment TRS criteria [9]. The underrepresentation of individuals with treatment-resistant psychiatric symptoms in studies similarly reduced statistical efficiency [10]. Another critical question in the TRS field is whether TRS represents a more severe form of schizophrenia, or if it represents a distinct subtype of schizophrenia with a different symptom profile and pathophysiology. Some clinical, imaging, biological, and genetic evidence supports the latter [11, 12]. Given the potentially complex genetic architecture of this trait and the discrepancies in the clinical definition of TRS, there remains disagreement on the best approach to maximize the informativity and power of genetic studies on TRS. Our work was designed as a proteomics analysis to identify functional brain proteins that distinguish TRS from schizophrenia, in an attempt to provide a new perspective on this issue by exploring the expression products.

Proteomic techniques are increasingly being used to screen for biomarkers of schizophrenia, while no studies have yet applied proteomics to TRS. Proteome-wide association study (PWAS) captures any variant affecting the coding regions of genes, and then assigns each protein-coding gene functional affecting scores, and is widely utilized to robustly prioritize candidate genes [13]. PWAS is a high-throughput approach where proteomic studies detect fewer expressed proteins than expressed genes detected by the transcriptome, but protein expression provides an accurate functional profile and reflects the complex interactions between genes and the environment, presenting an unbiased picture of the current physiological state. The importance of those interactions has been increasing in the research of neurological diseases [10]. We performed two independent PWAS to validate each other, and functional enrichment and annotation analysis was conducted based on the results. Colocalization analysis was performed to identify variant loci that were causally related to the expressed proteins. These methods may identify biomarkers and discern the specific mechanisms underlying TRS, thereby offering proof of its classification as a subtype of schizophrenia and facilitating the early detection of TRS.

## Materials and Methods

### GWAS summary data

The GWAS summary data of TRS and non-treatment-resistant schizophrenia (non-TRS) were derived from a recently published study [12]. Patients with TRS were derived from the CLOZUK1 and CLOZUK2 cohorts, with a total sample size of 10,501 individuals [14]. All patients with TRS were prescribed clozapine following at least two failed trials of antipsychotics and in accordance with National Institute for Health and Care Excellence guidelines for TRS. Patients with non-TRS were derived from 34 studies that were included in the meta-analysis by the Schizophrenia Working Group of the Psychiatric Genomics Consortium (PGC), with a total sample size of 20,325 patients with schizophrenia [15]. Fourteen studies with clinical records identified and excluded individuals with TRS, and cases from the remaining 20 studies without clinical records were conservatively included in our analysis as individuals with non-TRS. Owing to the number of different datasets and genotyping arrays involved in this analysis, the processing of TRS and non-TRS GWAS samples was performed separately on the data generated by the original study [16, 17]. Both of these imputations used the SHAPEIT/IMPUTE2 pipeline. Single Nucleotide Polymorphisms (SNPs) were restricted to minor allele frequencies (MAF) of 5% or higher and called in at least 20,000 combined samples, and any strand-ambiguous markers (A/T and G/C) with MAF ≥ 40% were discarded in both datasets.

### Human brain proteome reference weights for PWAS

Two human brain proteome reference weight datasets were obtained from recent publicly available studies. The discovery dataset was derived from Religious Orders Study and Rush Memory and Aging Project (ROS/MAP) cohorts, recruiting 391 individuals from two longitudinal clinical-pathologic cohort studies of aging and Alzheimer’s disease (AD) [18]. After quality control, 8,356 proteins from 376 subjects were included in our analysis. Among these, 262 were female and the average age at death was 89 years. The final clinical diagnosis included no cognitive impairment, mild cognitive impairment (MCI), AD dementia, or other causes of dementia. The confirmation dataset was derived from the Banner Sun Health Research Institute (Banner) containing 198 individuals [19]. After quality control, 11,518 proteins from 152 subjects were quantified. Of these, 87 were female and the average age at death was 85 years. Only individuals with a final diagnosis of AD or normal cognition were included. Both of the proteomic reference datasets utilized the same quality control procedures to identify and control the effects of clinical covariates (i.e., age, gender, and final clinical diagnosis of cognitive status) before estimating protein weights. Details can be found in the Supplementary aterial.

### Proteome-wide association study analysis

PWAS analysis was performed by integrating the TRS GWAS data with two brain proteomes using the FUSION pipeline (http://gusevlab.org/projects/fusion/). Briefly, we utilized FUSION to calculate protein weights in both the discovery and confirmation datasets individually. Subsequently, we combined the genetic effect of TRS (TRS GWAS *z*-score) with precomputed protein weights by calculating the linear sum of *z*-score × weight of independent SNPs to perform the PWAS of TRS. Only proteins identified in the discovery dataset and replicated in the confirmation dataset were considered TRS-associated proteins. The linkage disequilibrium reference panel routinely utilized 1,190,321 HapMap SNPs from 489 individuals of European descent from the 1000 Genomes Project in FUSION. We implemented 2,000 permutations to control the potential impact of multiple testing on PWAS results. The proteins with permutated *P* < 0.05 were considered significant. The design is presented in the Supplementary Figure S1.

### Colocalization analysis

Our research performed a colocalization analysis of all genes identified by the two-stage PWAS using the coloc R package. We evaluated the colocalization status of a gene by calculating the PP that the genetic and functional associations derived from a shared causal SNP (PP4). Genes with PP4 > 0.75 are considered to be colocalized.

### Functional enrichment and annotation analysis

The GO annotation and KEGG pathway enrichment analyses of the genes identified by PWAS were performed by the DAVID tool (https://david.ncifcrf.gov/). GO enrichment analysis includes biological process (BP), cellular component (CC), and molecular function (MF) analysis. KEGG database is a bioinformatics resource for mining significantly metabolic pathways enriched in the gene list. The purpose was to extract important GO terms and KEGG pathways, which can depict the characteristics of TRS. The results were considered statistically significant if *P* < 0.05.

## Results

### PWAS results of TRS

**Figure 1.**
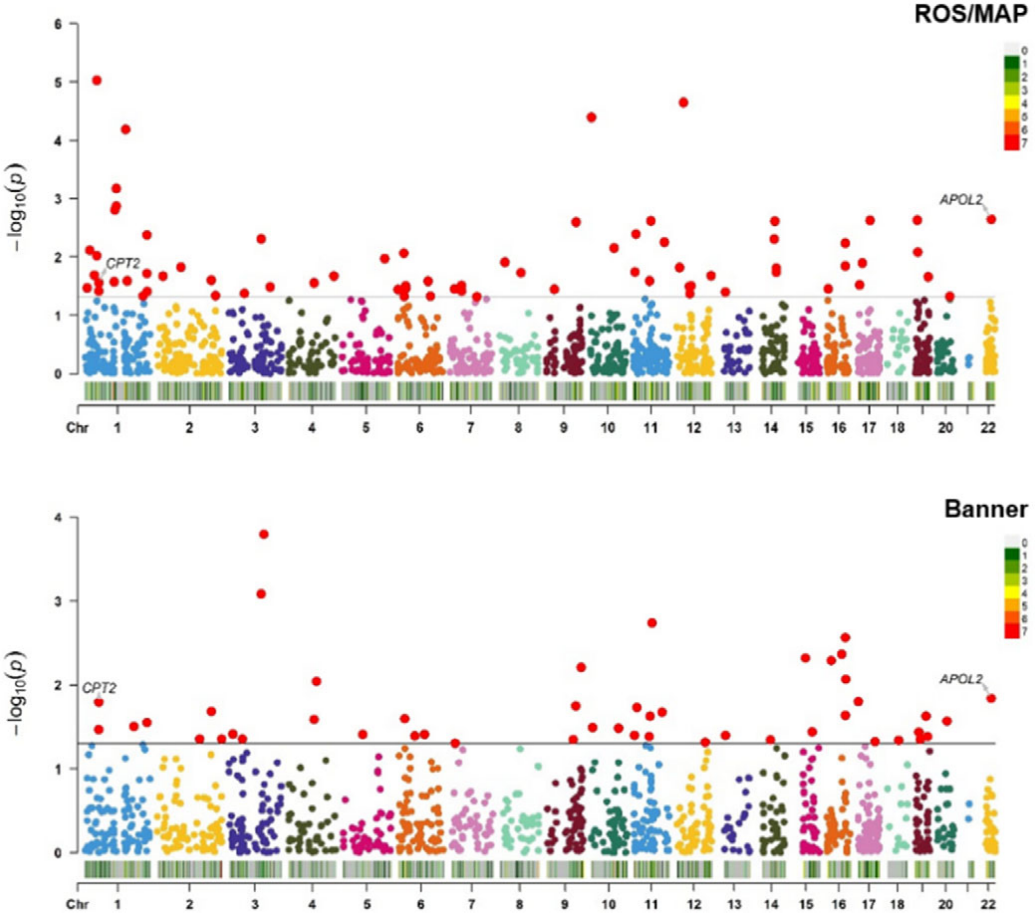
Manhattan plots of significant human brain proteins identified in proteome-wide association study (PWAS). Each point corresponds to a single test of association between a gene and phenotype, plotted according to genomic position on the x-axis and the strength of association (−log_10_ *P*-value) on the y-axis. Two common statistically significant proteins resulting from the analysis were mapped out.

In PWAS, two significant proteins were identified in the discovery reference dataset and replicated in the confirmation reference dataset, including CPT2 (*P*_PWAS-ROS/MAP_ = 4.15 × 10^−2^ and *P*_PWAS-Banner_ = 3.38 × 10^−3^) and APOL2 (*P*_PWAS-ROS/MAP_ = 4.49 × 10^−3^ and *P*_PWAS-Banner_ = 8.26 × 10^−3^). Specifically, in the discovery stage, a total of 24 TRS-related candidate proteins were identified using the ROS/MAP reference dataset, such as PRDX1 (*P*_PWAS-ROS/MAP_ = 1.00 × 10^−3^), APOL2 (*P*_PWAS-ROS/MAP_ = 4.49 × 10^−3^), and WARS2 (*P*_PWAS-ROS/MAP_ = 6.41 × 10^−3^). In the confirmation stage, a total of 19 TRS-related candidate proteins were identified using the Banner reference dataset, such as CPT2 (*P*_PWAS-Banner_ = 3.38 × 10^−3^) and ABCC1 (*P*_PWAS-Banner_ = 4.25 × 10^−3^). Statistically significant genes identified in PWAS analysis are shown in Table 1 and Figure 1.

**Table 1.** Significant proteins identified by PWAS.

Brain proteins		Chromosome	Permutation *p* value		COLOC.PP4
Symbol	Name		ROS/MAP	Banner	
*CPT2*	Carnitine Palmitoyltransferase 2	1	4.15 × 10^−2^	3.38 × 10^−2^	0.056
*APOL2*	Apolipoprotein L2	22	4.49 × 10^−3^	8.26 × 10^−3^	0.017
*CCDC91*	Coiled-Coil Domain Containing 91	12	2.27 × 10^−2^	–	0.981
*PRDX1*	Peroxiredoxin 1	1	1.00 × 10^−3^	–	0.894
*WARS2*	Tryptophanyl TRNA Synthetase 2	1	6.41 × 10^−3^	–	0.757
*FLAD1*	Flavin Adenine Dinucleotide Synthetase 1	1	2.94 × 10^−2^	–	0.065
*NIT1*	Nitrilase 1	1	1.26 × 10^−2^	–	0.055
*ICA1L*	Islet Cell Autoantigen 1 Like	2	3.11 × 10^−2^	–	0.042
*RABEP1*	Rabaptin, RAB GTPase Binding Effector Protein 1	17	4.03 × 10^−2^	–	0.036
*1-Mar*	Mitochondrial Amidoxime Reducing Component 1	1	4.21 × 10^−2^	–	0.025
*GANAB*	Glucosidase II Alpha Subunit	11	1.07 × 10^−2^	–	0.023
*CORO7*	Coronin 7	16	<0.0001	–	0.022
*TMEM25*	Transmembrane Protein 25	11	<0.0001	–	0.016
*FUT8*	Fucosyltransferase 8	14	<0.0001	–	0.014
*ALDH4A1*	Aldehyde Dehydrogenase 4 Family Member A1	1	3.79 × 10^−2^	–	0.012
*DBNL*	Drebrin Like	7	2.09 × 10^−2^	–	0.01
*LETMD1*	LETM1 Domain Containing 1	12	1.17 × 10^−2^	–	0.005
*TTC19*	Tetratricopeptide Repeat Domain 19	17	4.92 × 10^−2^	–	0.002
*RASA4B*	RAS P21 Protein Activator 4B	7	4.35 × 10^−2^	–	0.001
*ALAD*	Aminolevulinate Dehydratase	9	1.20 × 10^−3^	–	0.001
*TPP1*	Tripeptidyl Peptidase 1	11	9.24 × 10^−3^	–	0.001
*TUBA4A*	Tubulin Alpha 4a	2	3.23 × 10^−2^	–	<0.001
*NPM2*	Nucleophosmin/Nucleoplasmin 2	8	4.80 × 10^−2^	–	<0.001
*COQ10A*	Coenzyme Q10A	12	4.34 × 10^−3^	–	<0.001
*ABCC1*	ATP Binding Cassette Subfamily C Member 1	16	–	4.25 × 10^−3^	0.06
*DHODH*	Dihydroorotate Dehydrogenase	16	–	3.01 × 10^−2^	0.051
*MRVI1*	Murine Retrovirus Integration Site 1 Homolog	11	–	2.81 × 10^−2^	0.049
*ALDH5A1*	Aldehyde Dehydrogenase 5 Family Member A1	6	–	1.63 × 10^−2^	0.04
*COA7*	Cytochrome C Oxidase Assembly Factor 7	1	–	2.48 × 10^−2^	0.032
*ANKMY2*	Ankyrin Repeat And MYND Domain Containing 2	7	–	3.80 × 10^−2^	0.026
*TGM2*	Transglutaminase 2	20	–	4.11 × 10^−2^	0.008
*C1orf27*	Chromosome 1 Open Reading Frame 27	1	–	2.32 × 10^−2^	0.007
*GRIA4*	Glutamate Ionotropic Receptor AMPA Type Subunit 4	11	–	2.31 × 10^−2^	0.006
*GALK2*	Galactokinase 2	15	–	2.67 × 10^−2^	0.003
*TMEM245*	Transmembrane Protein 245	9	–	<0.0001	0.002
*KHDRBS2*	KH RNA Binding Domain Containing	6	–	2.03 × 10^−2^	0.001
*TMEM109*	Transmembrane Protein 109	11	–	2.00 × 10^−4^	0.001
*NUDT16*	Nudix Hydrolase 16	3	–	<0.0001	<0.001
*TMEM43*	Transmembrane Protein 43	3	–	<0.0001	<0.001
*PPA2*	Inorganic Pyrophosphatase 2	4	–	2.54 × 10^−2^	<0.001
*CRAT*	Carnitine O-Acetyltransferase	9	–	2.77 × 10^−2^	<0.001

*Note*: Only significant permutation *P* values are presented.

Abbreviation: PWAS, proteome-wide association study.

### Colocalization analysis

Of all 41 proteins identified by PWAS, colocalization analysis identified three genes that are causal for TRS and encoded functional proteins, including *CCDC91* (PP4 = 0.981), *PRDX1* (PP4 = 0.894), and *WARS2* (PP4 = 0.757). The results of the colocalization analysis of the genes identified by PWAS are presented in Table 1.

### Functional enrichment and annotation analysis

GO enrichment analysis of all 41 genes identified by PWAS results are shown in Table 2. DAVID detected 14 GO terms, such as mitochondrion (GO:0005739, *P* < 0.0001), Golgi apparatus (GO:0005794, *P* = 0.0145), oxidoreductase activity (GO:0016620, *P* = 0.0338), and protein domain specific binding (GO:0019904, *P* = 0.0117). For pathway enrichment analysis of the genes identified by PWAS, DAVID detected only one candidate pathway for TRS, metabolic pathways (hsa01100, *P* = 0.0312).

**Table 2.** GO enrichment analysis results of TRS-associated genes identified by PWAS.

Name	*P* value	Fold enrichment
GO:0006807 ~ nitrogen compound metabolic process	0.0244	78.5959
GO:0006491 ~ N-glycan processing	0.0330	57.9128
GO:0005739 ~ mitochondrion	0.0000	4.8738
GO:0005743 ~ mitochondrial inner membrane	0.0014	6.9664
GO:0005759 ~ mitochondrial matrix	0.0052	6.9069
GO:0005794 ~ Golgi apparatus	0.0145	3.3611
GO:0042470 ~ melanosome	0.0149	15.6914
GO:0070062 ~ extracellular exosome	0.0151	2.4289
GO:0008458 ~ carnitine O-octanoyltransferase activity	0.0054	358.3048
GO:0042802 ~ identical protein binding	0.0098	2.8254
GO:0019904 ~ protein domain specific binding	0.0117	8.1743
GO:0048039 ~ ubiquinone binding	0.0126	153.5592
GO:0003824 ~ catalytic activity	0.0237	12.2149
GO:0016620 ~ oxidoreductase activity, acting on the aldehyde or oxo group of donors, NAD or NADP as acceptor	0.0338	56.5744

Abbreviation: PWAS, proteome-wide association study.

## Discussion

In this work, we performed TRS-associated PWAS and found a total of 41 proteins that were differentially expressed, 2 of which were duplicated in the PWAS analysis of the discovery and confirmation dataset, suggesting a prominent role in the BPs of TRS, including CPT2 and APOL2. Owing to polygenic inheritance, a complex trait is often influenced by multiple genes with similar functions as annotated in gene pathways. We extended PWAS results from gene-based analysis to pathway-based analysis, identifying 14 GO terms and 1 candidate pathway for TRS. Aiming to understand the mechanisms driving GWAS risk loci, we performed a colocalization analysis of PWAS-identified genes and identified three variants that were causally related to expression in the human dorsolateral prefrontal cortex.

TRS may be associated with abnormalities in the β-oxidative metabolic pathway of long-chain fatty acids in mitochondria. We discovered a novel TRS-related protein CPT2. Carnitine palmitoyl transferases 2 (CPT2) is the core protein of a catalytically active multiprotein complex localized in the inner mitochondrial membrane, assisting long-chain fatty acids to enter the mitochondrial matrix for oxidation and energy. The role of the lipid regulatory system on TRS has been supported by studies. One work found that clozapine altered the activity of the AMPK-ACC-CPT1 pathway, a central pathway of lipid metabolism, to affect the lipid compositions of the neuronal membranes in the rat frontal cortex [20]. Abnormalities in membrane lipid composition have also been reported in the frontal cortex of patients with schizophrenia [21]. Beta-oxidation may be potentially linked to the pathogenesis of TRS. The metabolite of clozapine is capable of interacting with a wide range of neurotransmitter receptors, suggesting that TRS may have a neurobiological etiology [22]. One study found that deletion of CPT2 in the nervous system leads to elevated expression of β-oxidation enzymes [23]. Individuals with genetic disorders in mitochondrial fatty acid β-oxidation may suffer from neurological disorders, including seizures, encephalopathies, and cortical atrophy [24–26]. CPT activity is present in almost all brain regions especially the brainstem [27], and carnitine shuttle and β-oxidation genes are expressed primarily in astrocytes and neural stem cells [28], suggesting that CTP2 deficiency may involve the central nervous system. Our GO and pathway enrichment analysis also support the CTP2-centred bio-metabolic processes.

TRS may be linked to cholesterol transport and homeostasis. The other brain protein we identified was apolipoprotein L2 (APOL2). The apolipoprotein family of proteins facilitates the tightly regulated delivery of lipids and lipophilic substrates to specific cells in the brain, as well as regulating signal transduction pathways [29]. APOL2, mainly localized at the endoplasmic reticulum, is implicated in cholesterol biosynthesis and trafficking and is thought to mediate cell death induced by interferon-gamma or viral infection, indicating a role in inflammatory processes [30]. Dysregulation of the inflammatory response system has been associated with the pathophysiology of schizophrenia [31]. Prior works have found the gene *APOL2* was upregulated in the brains of schizophrenic patients, and polymorphisms in this gene were linked to schizophrenia risk [32]. Differential expression of *APOL2* has also been observed in individuals with substance use disorders, including cocaine, cannabis, and phencyclidine [33]. *APOL2* is highly expressed in some brain regions, including the hippocampus, intralobular white matter, and the medulla [34]. However, the biological function of APOL2 in the brain remains unclear.

Three gene variants identified by colocalization analysis implied a potential association with TRS pathogenesis, including *CCDC91*, *PRDX1*, and *WARS2.* *CCDC91* is highly expressed in the central nervous system, located in the nucleoplasm and trans-Golgi networks and is involved in subcellular transport and protein localization in the Golgi complex. One study showed an increased incidence of copy number variation in *CCDC91* in bipolar disorder patients [35]. Its interacting partners, GGA1 and GGA2, have been implicated in the pathophysiology of AD through interactions with β-amyloid precursors [36]. Also, our work identified one GO annotation associated with *CCDC91*: identical protein binding. PRDX is a protein family with antioxidant enzyme activity that reduces hydrogen peroxide and alkyl hydroperoxides in cells. Numerous studies have demonstrated the anti-inflammatory and anti-apoptotic effects of PRDX1 [37]. It appears to have neuroprotective activities in neuronal cells, which reduce reactive oxygen species-mediated cell death in schizophrenia [38]. Antipsychotic drugs affect *PRDX1* expression. *PRDX1* expression was increased in haloperidol-treated C6 cells but decreased in C6 cells treated with risperidone and clozapine [39]. *WARS2* encodes mitochondrial tryptophan-tRNA synthetase, a homologous class Ic enzyme. The clinical spectrum associated with *WARS2* defects appears to be quite broad, including clinical features (cardiomyopathy, movement disorders, retinitis pigmentosa, optic atrophy, hypoglycemia, etc.) as well as the age of onset and clinical course [40]. Also, *WARS2* mutations cause dopa-responsive early-onset parkinsonism and progressive myoclonus ataxia [41]. These works support the contention that the biparental loss-of-function *WARS2* variants cause mitochondrial dysfunction and disease. The effect of variants in these three genes on TRS needs further study.

Functional enrichment and annotation analysis revealed several mitochondrial-related results implicating mitochondrial function in the pathogenesis of TRS. Some evidence has confirmed that mitochondrial dysfunction is an important pathological factor in schizophrenia, including decreased mitochondrial respiration due to altered complex I activity [42], motor deficits, altered mitochondrial dynamics [43], increased levels of mitochondrial DNA mutations [44], and decreased cognitive abilities in mitochondrial diseases [45]. One study identified a strong correlation between a TRS susceptibility gene and mitochondrial dysfunction, which correlates with the dysregulation of NRG-1/mTOR/miR143-3p signaling [46]. More attention should be paid to the role of mitochondria in TRS.

Our results revolve more around lipid oxidation and inflammation, which tends to be, but is not fully explained by the “inflammation and oxidative stress” hypothesis of the neurobiological mechanisms of TRS. More evidence is being mined to support this hypothesis. A study showed elevated lipid peroxidation in patients with TRS compared to treatment-responsive schizophrenia patients and healthy controls. This exacerbated peroxidation process in TRS may reflect a deeper abnormality in the fatty acid content of synaptic membranes, leading to the dysfunction of neurons as well as its microenvironment [47]. Early neuroinflammation and chronic hyperactivation are thought to contribute to schizophrenia; high levels of inflammation may also play a role in treatment resistance [48]. There is no universally accepted or defined mechanism for TRS, limited by different criteria for TRS or small study sample sizes. Other hypotheses have been proposed regarding the neurobiological mechanisms of TRS, including differences in dopaminergic function, glutamate dysregulation, and serotonin dysregulation. Previous studies have provided genetic evidence for different hypotheses, indicating that TRS may develop through multiple pathways or change in potential mechanisms at specific times [49]. These theories are not mutually exclusive, and combining several pathways may contribute to the neurobiology of TRS [50]. These results are insufficient to highlight the distinctiveness of TRS from schizophrenia and more characteristic markers are needed to understand the BPs and prove the heterogeneity of TRS.

Our work is the first TRS-related study with proteomic analysis based on a large sample of GWAS summary data, providing an accurate functional profile that presents an unbiased picture of current physiological status. Despite this, the study has certain limitations. TRS is an underreported diagnosis, and although our definition of the phenotype is in line with international criteria, we acknowledge that there may still be individuals with treatment-resistant symptoms in non-TRS datasets. This could result in imperfect phenotypes and misclassifications that weaken our findings and reduce the exploratory power of the brain proteins analyzed. The study sample is mainly of European descent and the conclusions may not pertain to non-European countries. Furthermore, we performed a cross-sectional proteomics study, so no exploration of longitudinal changes in biomarkers was available. More research is needed to test our results in different patient populations and different phases of illness. Additionally, it is worth noting that the age and brain tissue preparation technique of the individuals included in the proteome reference datasets may have a slight impact on the final protein expression results. Finally, some proteins identified in the discovery of PWAS failed to be replicated in the confirmation PWAS, which is attributed to the limited sample size and the stochastic nature of high-throughput proteomic sequencing.

In conclusion, we performed PWAS analysis and identified two TRS-associated brain proteins, CPT2 and APOL2. Colocalization analysis based on PWAS results identified three variants that were causally related to protein expression, including *CCDC91*, *PRDX1*, and *WARS2.* Our results cautiously support that the pathological mechanism of TRS is linked to lipid oxidation and inflammation, where mitochondria-related functions may play a role.

## Data Availability

The datasets can be downloaded from the Psychiatric Genomics Consortium website (http://pgc.unc.edu).

## Supplementary material

For supplementary material accompanying this paper visit http://doi.org/10.1192/j.eurpsy.2023.20. click here to view supplementary material
