# Supplementary material for: Identification of novel functional brain proteins for treatment-resistant schizophrenia: Based on a proteome-wide association study
Source: Eur Psychiatry. 2023 Apr 14;66(1):e33. doi: 10.1192/j.eurpsy.2023.20 (PMC10228356; doi:10.1192/j.eurpsy.2023.20)
Supplement: Supplementary file 1 [file S0924933823000202sup001.docx]

**Materials and Methods**

***GWAS summary data***

TRS patients were derived from the CLOZUK1 and CLOZUK2 cohorts, with a total sample size of 10,501 individuals^1,2^. These individuals were prescribed clozapine in the UK after failing two trials of antipsychotics and were diagnosed with treatment-resistant schizophrenia (TRS) based on a history of taking clozapine, which has been validated in previous studies^3^. The use of a history of taking clozapine as equivalent to a research diagnosis of TRS has been validated in these samples^1^, as well as in independent studies^4,5^. Non-TRS patients were derived from 34 studies that were included in the meta-analysis by the Schizophrenia Working Group of the Psychiatric Genomics Consortium (PGC), with a total sample size of 20,325 individuals^6^. In 14 of these studies, clinical records were available to identify and remove all individuals with TRS. The remaining 20 studies were not screened as comparable data were not available, and therefore these samples were conservatively included in the analysis as non–treatment-resistant cases. For the identification of these sample without individual-level medical records, the categorization of a recently published authoritative paper was used, which validated the reliability of TRS results under this classification using two independent cohorts. The details of the dataset of non-TRS GWAS samples included in the PGC are shown in the following Table S1.

***Proteome reference datasets preparation***

The brain autopsy procedures for ROSMAP dataset are as follows^7,8^. “As a condition of entry, participants signed an anatomical gift act donating their entire brain and spinal cord, and selected portions of nerve and muscle to investigators for studies of aging and AD. Three staff members are on-call 24 h per day, 7 days a week. The study coordinator works with the family, facility, and funeral home to get the body to the morgue, and arranges for two technicians to meet the body, one to remove the cerebrospinal fluid and brain, and the second to remove the spinal cord, nerve, and muscle. After removal of the brain, the brain is weighed, and the brainstem and cerebellum are removed. The brain and cerebellum are cut into 1-cm slabs on a Plexiglas jig. Digital pictures of the whole brain and slabs are obtained after macroscopic evaluation. One half of the brain, one half of the cerebellum, and a portion of the midbrain are frozen in a –80°C freezer for biochemical studies. The remainder of the brain is placed in 4% paraformaldehyde. After 3 days of paraformaldehyde fixation, blocks are dissected from specified brain regions (including the hippocampus, entorhinal cortex, cingulate cortex, midfrontal cortex, superior frontal cortex, inferior temporal cortex, middle temporal cortex, inferior parietal cortex, primary occipital cortex, basal ganglia, thalamus, and midbrain) and paraffin embedded for subsequent histochemistry and immunocytochemistry. Blocks from all areas of visible pathology (e.g., infarctions, tumors) are also obtained. The remainder of the brain is transferred to a graded cryoprotectant solution (final solution of 20% glycerol and 2% DMSO in phosphate buffer) for long-term storage at 4°C.

The entire spinal cord is removed. Small transverse sections from the cervical, thoracic, and lumbar segments are frozen in a –80°C freezer for biochemical studies, embedded in paraffin for histochemistry and immunocytochemistry, and fixed in glutaraldehyde for electron microscopy. The remainder of the spinal cord is transferred to cryoprotectant solution for long-term storage at 4°C. A 1-cm section of each sciatic nerve is dissected from the popliteal fossa and is stored in glutaraldehyde for plastic embedding, formalin for paraffin embedding, and the remainder is stored in cryoprotectant. Two 1-cm blocks are dissected from the belly of the deltoid, quadriceps femoris, and gastrocnemius muscles bilaterally. One block is placed in OCT and snap frozen in liquid nitrogen insulated by isopentane and stored at –80°C for biochemical studies and histochemistry, and the remainder is fixed in 4% paraformaldehyde for 3 days after which a portion is embedded in paraffin.”

The brain autopsy procedures for Banner dataset are as follows^9^. “The olfactory bulbs and tracts as well as the pineal and pituitary glands are removed and the cerebellum and brainstem are severed from the cerebrum by a transverse cut across the rostral pons, keeping the entire substantia nigra (SN) with the coronally sectioned cerebral slices. The leptomeninges are stripped from both cerebrum and cerebellum for storage as research material. The cerebellum is separated from the brainstem by severing the cerebellar peduncles. Each hemicerebellum is sliced into 4–5 segments in the parasagittal plane. The brainstem is sliced into left and right halves. The cerebrum is sliced into 1 cm segments in the coronal plane. The left side slices are used for immersion fixation while the right-side slices are rapidly frozen between sheets of dry ice (20 × 20 × 3 cm).”

***Proteome reference datasets population***

***Discovery human brain proteome reference dataset ROSMAP***

The participants in ROSMAP were older adults without dementia who agreed to participate in detailed longitudinal clinical evaluations and organ donation. After quality control, 376 individuals with both proteomic and genetic data were included in the discovery analysis^10^. The participants were 262 females and the average age at death was 89 years old. The final clinical diagnosis of cognitive status was determined by a neurologist with expertise in dementia using all available clinical data, but without knowledge of postmortem data. The diagnoses included no cognitive impairment, mild cognitive impairment (MCI), Alzheimer’s disease (AD) dementia, or dementia due to other causes. The diagnosis was based on the guidelines of the National Institute of Neurological and Communicative Disorders and Stroke and the AD and Related Disorders Association, and MCI was based on accepted criteria. Case conferences including one or more neurologists and a neuropsychologist were used for consensus as necessary.

Proteomic profiling was performed using isobaric tandem mass tag (TMT) peptide labeling. Samples were randomized by various factors (age, sex, postmortem interval, cognitive diagnosis, and pathologies into 50 batches) before TMT labeling to minimize batch effects. After protein quantification, regression was used to remove the effects of proteomic sequencing batch, mass spectrometry reporter quantification mode, sex, age at death, postmortem interval, study (ROS versus MAP) and the final clinical diagnosis of cognitive status from the proteomic profile.

***Conformation human brain proteome reference dataset Banner***

The participants in the Banner study or their legal representatives signed an informed consent form, and the study was approved by the institutional review board of the Banner Sun Health Research Institute. After quality control, 152 individuals with both proteomic and genetic data were included in the replication analysis^10^. These participants were 87 females and the average age at death was 85 years old. Participants consented to annual standardized medical, neurological and neuropsychological testing. Research diagnoses were made using approved research guidelines and a final clinicopathological diagnosis was made after reviewing all clinical, medical records and neuropathological findings. Only individuals with a final diagnosis of normal cognition or AD were included in the proteomic analysis.

The confirmation human brain proteomes were profiled from the dPFC of postmortem brain samples using the same proteomic analysis procedure as described for the ROSMAP dataset. The same quality control procedures were applied to remove proteins with more than 50% missing data, identify and remove outliers, and remove the effects of clinical covariates (that is, sex, age, final clinical diagnosis of cognitive status) before estimating protein weights. More details about the sample description, proteomic analysis, quality control, and statistical analyses are provided in the original paper^10^.


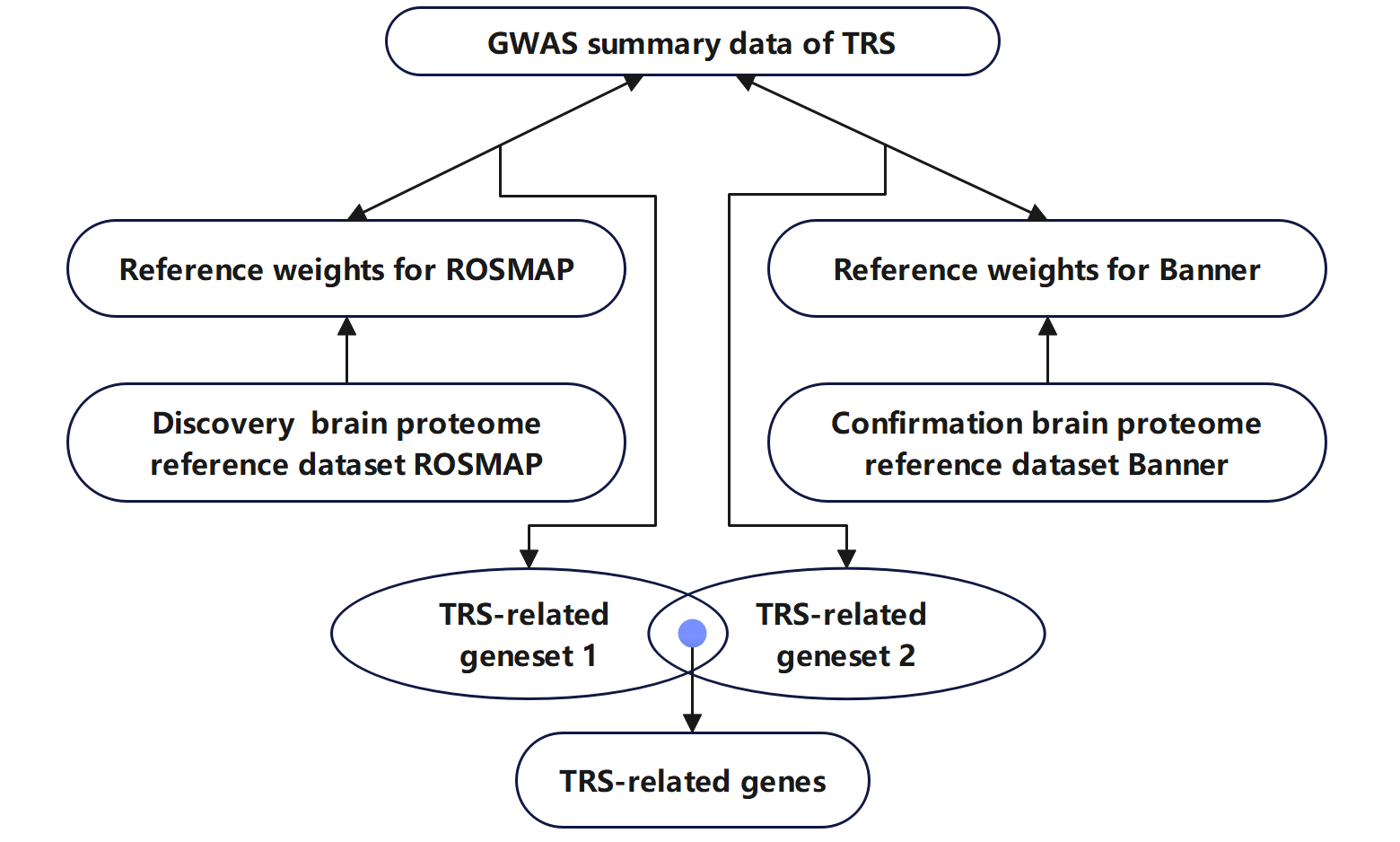


**Figure S1. Flowchart of the study design**

**Table S1.Datasets included in the PGC non-TRS GWAS sample**

| DATASET ID* | TRS CASES (EXCLUDED)** | NON‐TRS CASES |
| --- | --- | --- |
| PORT | 22 | 328 |
| TOP8 | 25 | 351 |
| ABER | 29 | 691 |
| ASRB | 33 | 476 |
| DUBL | 38 | 234 |
| SWE1 | 60 | 161 |
| IRWT | 78 | 1222 |
| DENM | 105 | 387 |
| UCLO | 134 | 386 |
| MUNC | 166 | 271 |
| SWE6 | 228 | 865 |
| BOCO | 289 | 1558 |
| S234 | 402 | 1675 |
| SWE5 | 433 | 1368 |
| AJSZ | n/a | 896 |
| BULS | n/a | 527 |
| CATI | n/a | 409 |
| CAWS | n/a | 476 |
| CIMS | n/a | 71 |
| EDIN | n/a | 368 |
| EGCU | n/a | 239 |
| ERSW | n/a | 322 |
| GRAS | n/a | 1086 |
| LACW | n/a | 157 |
| LIE2 | n/a | 137 |
| LIE5 | n/a | 509 |
| MGS2 | n/a | 2681 |
| MSAF | n/a | 327 |
| PEWB | n/a | 597 |
| PEWS | n/a | 82 |
| UCLA | n/a | 705 |
| UMEB | n/a | 375 |
| UMES | n/a | 197 |
| ZHH1 | n/a | 191 |

* For full details see Schizophrenia Working Group of the Psychiatric Genomics Consortium (2014). ** TRS ascertainment could not be carried out in datasets marked with "n/a", as individual‐level clinical records were not available.

1 Pardinas, A. F. *et al.* Common schizophrenia alleles are enriched in mutation-intolerant genes and in regions under strong background selection. *Nat Genet* **50**, 381-+, doi:10.1038/s41588-018-0059-2 (2018).

2 Hamshere, M. L. *et al.* Genome-wide significant associations in schizophrenia to ITIH3/4, CACNA1C and SDCCAG8, and extensive replication of associations reported by the Schizophrenia PGC. *Mol Psychiatry* **18**, 708-712, doi:10.1038/mp.2012.67 (2013).

3 Mortimer, A. M., Singh, P., Shepherd, C. J. & Puthiryackal, J. Clozapine for treatment-resistant schizophrenia: National Institute of Clinical Excellence (NICE) guidance in the real world. *Clin Schizophr Relat Psychoses* **4**, 49-55, doi:10.3371/CSRP.4.1.4 (2010).

4 Ucok, A. *et al.* Correlates of Clozapine Use after a First Episode of Schizophrenia: Results From a Long-term Prospective Study. *CNS Drugs* **30**, 997-1006, doi:10.1007/s40263-016-0358-z (2016).

5 Suzuki, T. *et al.* Defining treatment-resistant schizophrenia and response to antipsychotics: a review and recommendation. *Psychiatry Res* **197**, 1-6, doi:10.1016/j.psychres.2012.02.013 (2012).

6 Schizophrenia Working Group of the Psychiatric Genomics, C. Biological insights from 108 schizophrenia-associated genetic loci. *Nature* **511**, 421-427, doi:10.1038/nature13595 (2014).

7 Schneider, J. A., Arvanitakis, Z., Leurgans, S. E. & Bennett, D. A. The neuropathology of probable Alzheimer disease and mild cognitive impairment. *Ann Neurol* **66**, 200-208, doi:10.1002/ana.21706 (2009).

8 Bennett, D. A. *et al.* The Rush Memory and Aging Project: study design and baseline characteristics of the study cohort. *Neuroepidemiology* **25**, 163-175, doi:10.1159/000087446 (2005).

9 Beach, T. G. *et al.* Arizona Study of Aging and Neurodegenerative Disorders and Brain and Body Donation Program. *Neuropathology* **35**, 354-389, doi:10.1111/neup.12189 (2015).

10 Wingo, A. P. *et al.* Integrating human brain proteomes with genome-wide association data implicates new proteins in Alzheimer's disease pathogenesis. *Nat Genet* **53**, 143-+, doi:10.1038/s41588-020-00773-z (2021).
